# SARS-CoV-2 antibodies in dogs and cats in a highly infected area of Brazil during the pandemic

**DOI:** 10.3389/fvets.2023.1111728

**Published:** 2023-02-23

**Authors:** Samar Afif Jarrah, Louise Bach Kmetiuk, Fabrizia Valleriani, Barbara Bonfini, Alessio Lorusso, Violetta Vasinioti, Nicola Decaro, Marco Tulio dos Santos, Kledir Anderson Hofstaetter Spohr, Annamaria Pratelli, Anna Serroni, Sara Capista, Valéria Regia Franco Sousa, Alexander Welker Biondo, Luciano Nakazato, Valéria Dutra

**Affiliations:** ^1^Laboratory of Molecular Biology, Federal University of Mato Grosso, Cuiabá, MT, Brazil; ^2^Department of Veterinary Medicine, Federal University of Paraná, Curitiba, PR, Brazil; ^3^Department of Virology, Istituto Zooprofilattico Sperimentale dell'Abruzzo e del Molise “G. Caporale”, Teramo, TE, Italy; ^4^Department of Veterinary Medicine, University of Bari, Valenzano, BA, Italy

**Keywords:** Brazil, companion animals, pets, serosurvey, coronavirus, SARS-CoV-2

## Abstract

SARS-CoV-2 was a worldwide threat during the COVID-19 pandemic, and the state of Mato Grosso had the second highest mortality rate in Brazil, with 427. 4 deaths/100,000 inhabitants. However, no large-scale study among dogs and cats in such highly infected areas of Brazil has so far been conducted. Accordingly, the present study reports on a serosurvey among dogs and cats in Cuiabá, capital of Mato Grosso from November 2020 to July 2021, where the human mortality rate was 605/100,000 at that time. Overall, 33/762 dogs (4.3%) and 4/182 cats (2.2%) were found to be seropositive for SARS-CoV-2 through ELISA, and 3/762 dogs (0.4%) and 3/182 cats (1.6%) were seropositive through the serum neutralization test. Cats presented higher seroprevalence with higher titers of neutralizing antibodies. Although N-protein based ELISA may be a good screening test, cross-reactivity with other canine coronaviruses may impair its diagnostic use among dogs.

## 1. Introduction

Severe acute respiratory syndrome coronavirus 2 (SARS-CoV-2) was identified in Wuhan, China, at the end of 2019, as the cause of the coronavirus disease 2019 (COVID-19), which led to a pandemic scenario in early 2020 (1). Pneumonia and acute respiratory distress syndrome were the main clinical human signs of COVID-19 (2).

As the pandemic spread, reports of dogs and cats that were naturally infected by SARS-CoV-2 soon emerged worldwide. These animals were generally asymptomatic and their cases were associated with infected owners (3). Investigations on animal susceptibility to SARS-CoV-2 showed that cats intranasally inoculated with high doses of SARS-CoV-2 were able to infect other cats by means of airborne aerosols. Infected cats presented neutralizing antibodies and lesions in their respiratory tract but no clinical signs. They were more susceptible to SARS-CoV-2 infection than dogs (4). These findings have highlighted the reverse zoonotic potential of the disease cycle between owners and their pets, considering that outdoor access may have exposed dogs and cats to a contaminated environment. Several reports on experimental and natural infection with SARS-CoV-2 among animals have been published, with confirmation of these animals' susceptibility, regarding dogs (4–6), cats (4, 5, 7–12), ferrets (4, 6, 9), hamsters (13), non-human primates (14–17), and bats (18).

Cuiaba, the capital of the state of Mato Grosso, in central Brazil, has been severely affected by the COVID-19 pandemic. There were 48,152 confirmed cases in 2020, 67,548 in 2021 and 132,667 up to May 2022, out of a total population of 612,547 inhabitants. This state presented the second highest nationwide COVID-19 mortality rate, with 427.4 deaths/100,000 inhabitants. In the state capital, the rate was 605 deaths/100,000 habitants (19, 20). The first report from Brazil of a pet infected with SARS-CoV-2 also came from Cuiabá (12). Furthermore, also in Mato Grosso, a free-ranging black-tailed marmoset (*Mico melanurus*) that had been hit by a car was also found to be infected with this virus (21). These reports thus showed the impact of this highly contaminated environment on natural infection among domestic and wild animal species.

The first large-scale study on companion animals anywhere in the world was conducted in Italy and showed that 15/451 dogs (3.3%) and 11/191 cats (5.8%) presented SARS-CoV-2 neutralizing antibodies, but that there were no RT-PCR positive samples (22). Another study conducted in Texas, USA, showed that 3/17 cats (17.6%) and 1/59 dogs (1.7%) were positive for SARS-CoV-2 through RT-PCR and that 7/16 cats (43.8%) and 7/59 dogs (11.9%) presented neutralizing antibodies (23). Recently, 9/29 cats (31.0%) and 4/10 dogs (40.0%) in Rio de Janeiro were found to be positive through RT-PCR or serum neutralization (24). Higher prevalence was observed among pets whose owners were positive for COVID-19, which suggested that human contact may be a determining factor for infections among dogs and cats (23–26).

Despite the worldwide threat posed by SARS-CoV-2 during the pandemic, in which the state of Mato Grosso had the second highest mortality rate in Brazil, no large-scale study on dogs and cats in highly infected areas of Brazil has so far been conducted. Accordingly, the present study reports on a serosurvey among dogs and cats in Cuiabá, the state capital of Mato Grosso, from November 2020 to July 2021, where the human COVID-19 mortality rate was very high at that time.

## 2. Method

### 2.1. Sampling

This study was based on blood sampling collected according to convenience at the Veterinary Teaching Hospital (VTH) of the Federal University of Mato Grosso, in central Brazil, between November 2020 and July 2021. The animals brought to the VTH for attendance were mostly from the cities of Cuiaba and Varzea Grande. No restrictions were imposed on the sampling, with regard to medical history, age, breed, vaccination status, owner medical history or reason for attendance. After serological results, owners of seropositive pets were contacted about their SARS-CoV-2 infection status at the time of sampling.

In total, serum samples from 762 dogs (296 in November–December 2020 and 466 in January–July 2021) and 182 cats (68 in November–December 2020 and 114 in January–March 2021) were collected. These were placed in cryovial tubes and were immediately sent in dry ice to the Department of Veterinary Medicine, University of Bari, Valenzano, Italy, and to the Istituto Zooprofilattico Sperimentale dell'Abruzzo e del Molise “G. Caporale”, Teramo, Italy, for detection of SARS-CoV-2 antibodies. Serum samples were analyzed by means of ELISA to detect antibodies against the SARS-CoV-2 nucleocapsid (N) protein, and through the serum neutralization (SN) assay to assess presence of SARS-CoV-2 neutralizing antibodies. One positive and one negative control serum sample were kindly provided by the Istituto Nazionale Malattie Infettive “Lazzaro Spallanzani” (INMI, Rome, Italy) and were included in the assays. The B.1 SARS-CoV-2 isolate was used as the reference strain, and the viral titer of the stock was determined by means of the TCID50 assay, as previously described (27). The B.1 lineage strain (virus name: hCoV-19/Italy/ABR-IZSGC-TE46419/2020, accession ID: EPI_ISL_529023) has been a human isolate adapted on VERO E6 cells (fourth passage), first identified by qRT-PCR on human nasopharyngeal swab (28), and then by high-throughput sequencing to obtain the whole genome sequence (29). Viral isolation was performed on VERO E6 cells under biosafety level 3 (BSL-3) conditions. The B.1 strain was propagated into VERO E6 cells using MEM supplemented with 10% FBS. Cells were seeded in 175 cm2 flasks at 106 cells/mL and after 24 h were infected with 5 mL of a viral suspension at 0.01 multiplicity of infection. The flasks were incubated at 37°C in a humidified atmosphere of 5% CO_2_ and observed daily under an inverted optical microscope. When cytopathic effect (CPE) affected 80–90% of the cell monolayer, the supernatant was collected and centrifuged at 4°C 2,000 rpm for 10 min to remove the cellular pellet. Then, the supernatant was aliquoted and stored at −80°C. Before use, the virus was titrated in serial 1 log dilutions (from 1 log to 8 log) in 96-well culture plates of Vero E6 cells to determine the 50% tissue culture infective dose (TCID50). Plates were incubated at 37°C and checked every day to identify CPE using an inverted optical microscope. The endpoint titers were calculated according to the Reed and Muench method based on 10 replicates for titration (30).

### 2.2. Cell culture

The grivet monkey (*Cercopithecus aethiops*) kidney epithelial cell line Vero E6 (C1008) was kindly provided by the INMI. The cells were maintained in minimal essential medium (MEM, Sigma Aldrich, Merck Life Science S.r.l., Milan, Italy), supplemented with 10% fetal bovine serum (FBS, Sigma Aldrich, Merck Life Science S.r.l., Milan, Italy), 106 IU/L of penicillin, 10 g/L of streptomycin, 5 × 106 IU/L of nystatin and 125 mg/L of gentamicin (IZSAM). The cell line was regularly checked for mycoplasma contamination, and its absence was verified through PCR (Mycoplasma Detection Testing, Thermo Fisher, Waltham, MA, USA).

### 2.3. Enzyme-linked immunosorbent assay

A double-antigen ELISA kit was used for detection of specific antibodies against the SARS-CoV-2 nucleocapsid protein in animal serum samples. Specific IgG antibodies binding to the SARS-CoV-2 N protein were determined using ERADIKIT COVID19-IgG (cat: 26867-02; In3diagnostic, Turin, Italy). The results were defined based on the calculated ratio described in the following formula and were expressed as percentages: PR (%) = (OD test sample–OD negative control)/(OD positive control–OD negative control). Values ≥ 40% were considered positive for the presence of antibodies against SARS-CoV-2.

### 2.4. Serum neutralization assay

A previously described SN assay (27) was applied to assess the presence of neutralizing antibodies against SARS-CoV-2 in dog and cat serum samples. Before testing, the serum samples were inactivated by heating at 56°C for 30 min. Twofold serial dilutions (from 1:10 to 1:1280) of the tested samples and the positive and negative control samples were prepared in 96-well plates using MEM supplemented with 2% FBS. Negative control serum samples included in the analysis were of human and canine/feline origin, one for each species and collected before SARS-CoV-2 emergence. Positive serum samples were of human and canine/feline origin as previously described (31, 32). Serum samples from dogs and cats tested negative also for alpha coronaviruses by neutralization assays. Subsequently, an equal volume of 100 TCID 50/mL of viral isolate was added to the diluted serum samples, and the plates were incubated for 30 min at 37°C in 5% CO_2_. After incubation, the serum-virus solutions were transferred to 96-well plates containing confluent Vero E6 cells that had been seeded on the previous day. These plates were incubated for 72 h at 37°C in 5% CO_2_ and were observed using an inverted microscope for detection of any virus-specific cytopathic effect (CPE). The neutralization titer was defined as the reciprocal of the highest dilution without any CPE in the wells, and the positive threshold was set at 1:10.

### 2.5. Ethics statement

This project was approved by the Ethics Committee on Animal Use at the Federal University of Mato Grosso (protocol number 23108.043344/2020-62).

## 3. Results

Overall, the ELISA test revealed that samples from 33/762 dogs (4.3%) dogs and 4/182 cats (2.2%) were seropositive, while the SN assay showed that samples from 3/762 dogs (0.39%) (SN titers ranging from 1:20 to 1:40) and 3/182 cats (1.64%) (SN titers ranging from 1:40 to 1:320) were seropositive. Information on all the SN-positive pets was gathered and is presented in Table 1. The cat owners did not always provide information about when their own SARS-CoV-2 infection started. Only one dog (ID 418) presented a history of respiratory disorder. All the seropositive pets were from households in which the owner was positive for SARS-CoV-2 at the time of sampling. The dog owner with the highest neutralizing antibody titer reported having contracted SARS-CoV-2 infection 1 month before the dog was sampled.

**Table 1:** Epidemiological data on dogs and cats that were seropositive for SARS-CoV-2 through serum neutralization (SN) assays.

**ID**	**Sp**	**Sex**	**Breed**	**Age**	**Sampling date**	**History**	**ELISA**	**SN**	**Street access**	**COVID-19 household status**
397	Dog	F	Basset	3 Y	May 25, 2021	Idiopathic epilepsy	24.00%	1:20	No	Yes, January 2021
418	Dog	F	Shih tzu	11 M	Jan 28, 2021	Sneezing, nasal secretion	33.81%	1:10	Yes	Yes, November 2021
688	Dog	M	Shih tzu	7 Y	May 20, 2021	Acute renal insufficiency	40.0%	1:40	No	Yes, April 2021
5	Cat	M	Mixed breed	8 M	Dec 16, 2020	Vomiting, intense dehydration, dyschezia, dysuria, anorexia, and weight loss	41.60%	1:320	Yes	COVID-19 symptoms; infection not confirmed
70	Cat	F	Siamese	4 Y	Feb 2021	Urinary infection	55.19%	1:80	No	Yes, mid-2020
93	Cat	F	Mixed breed	1 Y	Apr 11, 2021	Fracture	45.00%	1:40	Yes	Yes, date unknown
121	Cat	F	Mixed breed	2 Y	Jan 8, 2021	Elective castration	31.96%	Negative (< 1:10)	No	Yes, 2020

## 4. Discussion

This study showed that SARS-CoV-2 antibodies were present in serum samples from dogs and cats attended at the Veterinary Teaching Hospital, in the same way as reported previously in several other studies worldwide (4, 6, 11, 22–24, 26, 33–35). In brief, these studies were conducted during the pandemic and reported occurrences of infection among cats and dogs through positive PCR test results, sometimes in the presence of clinical signs, along with presence of SARS-CoV-2 neutralizing antibodies. These previous results were obtained both from owned pets that had been kept indoors and from stray animals.

Since most previous studies also used ELISA as the screening test in serological surveys (25, 31, 35, 36), the discrepancy found in the present study between ELISA and SN findings from dogs may have been due to cross-reactions with other endemic coronaviruses (25, 35). Only 3 out of 33 ELISA-positive canine serum samples were also positive through SN. Not surprisingly, the SARS-CoV-2 N protein has been associated with cross-reactions with other animal coronaviruses such as the feline coronavirus (FCoV) and the canine coronavirus (CCoV). Moreover, the N protein has been considered to be a conserved viral protein, sharing common antigenic epitopes related to SARS-CoV-2 (35). In addition, previous studies have suggested that SARS-CoV-2 infection was not very efficient to elicit N protein specific antibodies (37). Lastly, the high proportion of SN-negative dogs that tested positive through ELISA may be explained by a milder course of infection, compared with cats (6, 38). As the study herein was limited to only an ELISA assay detecting N-protein, future studies should include at least one major surface protein, such as receptor binding site, spike protein, or trimeric spike proteins. Accordingly, higher human levels of neutralizing antibodies have been correlated with severe illness (39). In contrast to what was observed in the dogs of the present study, feline serum samples displayed higher agreement between ELISA and SN results, since out of four cat samples that were positive through ELISA, three of them also tested positive through SN. These results corroborated the results from previous studies on cats, in which higher seropositivity rates were observed and higher neutralizing antibody titers were developed than among dogs (6, 26, 31, 40).

A SARS-CoV-2 serosurvey study in France showed that only 5/449 dogs (1.1%) tested positive through ELISA before the emergence of the virus, while 25/443 dogs (5.5%) displayed antibodies during the COVID-19 pandemic. Seroconversion was observed in the cases of at least 8/218 dogs (3.7%) that were sampled twice during the survey period (25). Also in that study, dogs showed low susceptibility to SARS-CoV-2, such that viral transmission from and between dogs was weak or absent. However, comparative high seropositivity to SARS-CoV-2 was reported in 73/388 (18.9%) cats and 39/243 (16.0%) dogs from three veterinary clinics of Poland (33). This seropositivity was probably biased due to sampling dogs and cats with owner concerns and veterinary care and high infection rate in human population during the fourth SARS-CoV-2 wave in Poland (33). In the present study, samples were collected over a long period of time with different SARS-CoV-2 strains and epidemiological situations, which may have influenced both owner and pet seropositivity.

Lastly, pets sharing the household with a SARS-CoV-2-infected owner were found to present higher risk of infection in a previous study (26). All the pets presenting neutralizing antibodies in the present study were living in COVID-19-positive households. This confirms that pet owners constitute an associated risk factor for infection among their pets. As previously suggested, omicron transmission may occur by back-and-forth, and thus human re-infection with other novel viral strains detected in animals should be consider (41, 42). In such scenario, SARS-CoV-2 infection in humans and animals should always include sequencing to identify novel strains for passive and active immunotherapy development (41–43).

The present study showed that dogs and cats in areas of widespread human COVID-19 presented low rates of occurrence of SARS-CoV-2 antibodies. Positive pets mostly displayed non-specific mild clinical signs. Considering that positive pets were associated with COVID-19-positive households, infected owners should take the same precautions regarding isolation from their pets as they should do in relation to other people. The present study was the first large-scale serological survey on SARS-CoV-2 among pets carried out in central Brazil and was conducted in the area with the second highest COVID-19 human mortality rate nationwide.

## Data availability statement

The original contributions presented in the study are included in the article/supplementary material, further inquiries can be directed to the corresponding author.

## Ethics statement

This project was approved by the Ethics Committee on Animal Use at the Federal University of Mato Grosso (Protocol No. 23108.043344/2020-62). Written informed consent was obtained from the owners for the participation of their animals in this study.

## Author contributions

LN and VD contributed to the conception and design of the study. SJ, MdS, KS, AP, VS, AB, LN, and VD performed field work and sample processing. FV, AS, SC, BB, AL, VV, and ND performed serological analyses. SJ, LK, AB, LN, and VD wrote the first draft of the manuscript. SJ, LK, FV, BB, AL, VV, ND, MdS, KS, AS, SC, AP, VS, AB, LN, and VD wrote sections of the manuscript. All authors contributed to the manuscript revision and read and approved the submitted version.
